# Minneapolis in September, 1902

**Published:** 1902-06

**Authors:** 


### MINNEAPOLIS IN SEPTEMBER, 1902.

Plans are rapidly going forward all over the country to make the coming meeting a phenomenal success. The favored railroad rates already announced for the convention have awakened interest in many centres over the country. The Journal will again be active in assisting those in the East to reach Minneapolis. It further desires that veterinarians from all over the country whose route may be via Chicago to assemble in that city on Sunday, August 31st, and go in one body by the "Burlington" route to Minneapolis. The many courtesies enjoyed at the hands of this company at Omaha and Des Moines have made it a popular line among the profession, and every agent of this well-known line has received special instructions to look after the veterinarians in September. The JOURNAL will be glad to afford any information desired, secure transportation, arrange for berths, and all else for the comfort of those contemplating attendance.

